# Lethal Mutagenesis of Rift Valley Fever Virus Induced by Favipiravir

**DOI:** 10.1128/AAC.00669-19

**Published:** 2019-07-25

**Authors:** Belén Borrego, Ana I. de Ávila, Esteban Domingo, Alejandro Brun

**Affiliations:** a Centro de Investigación en Sanidad Animal (CISA-INIA), Madrid, Spain; b Centro de Biología Molecular “Severo Ochoa” (CSIC-UAM), Madrid, Spain; c Centro de Investigación Biomédica en Red de Enfermedades Hepáticas y Digestivas (CIBERehd) del Instituto de Salud Carlos III, Madrid, Spain

**Keywords:** favipiravir, Rift Valley fever virus, T-705, lethal mutagenesis

## Abstract

Rift Valley fever virus (RVFV) is an emerging, mosquito-borne, zoonotic pathogen with recurrent outbreaks taking a considerable toll in human deaths in many African countries, for which no effective treatment is available. In cell culture studies and with laboratory animal models, the nucleoside analogue favipiravir (T-705) has demonstrated great potential for the treatment of several seasonal, chronic, and emerging RNA virus infections in humans, suggesting applicability to control some viral outbreaks. Treatment with favipiravir was shown to reduce the infectivity of Rift Valley fever virus both in cell cultures and in experimental animal models, but the mechanism of this protective effect is not understood. In this work, we show that favipiravir at concentrations well below the toxicity threshold estimated for cells is able to extinguish RVFV from infected cell cultures. Nucleotide sequence analysis has documented RVFV mutagenesis associated with virus extinction, with a significant increase in G to A and C to U transition frequencies and a decrease of specific infectivity, hallmarks of lethal mutagenesis.

## Abstract (precis)

Rift Valley fever virus (RVFV) is an emerging, mosquito-borne, zoonotic pathogen with recurrent outbreaks taking a considerable toll in human deaths in many African countries, for which no effective treatment is available. In cell culture studies and with laboratory animal models, the nucleoside analogue favipiravir (T-705) has demonstrated great potential for the treatment of several seasonal, chronic, and emerging RNA virus infections in humans, suggesting applicability to control some viral outbreaks.

## INTRODUCTION

Rift Valley fever virus (RVFV) is a mosquito-borne bunyavirus belonging to the recently reclassified *Phlebovirus* genus (family, *Phenuiviridae*; order, *Bunyavirales*). RVFV causes an important disease in ruminants that is often transmitted to humans after the occurrence of epizootic outbreaks. Although the disease has been reported only in African countries with some incursions in the Middle East and Indian Ocean islands, there are concerns for its potential spread to other locations, including Europe (1, 2). Currently, there is no available treatment or licensed Rift Valley fever (RVF) vaccine in Europe; therefore, the development of effective control strategies is an essential field of research. The use of antiviral agents for treatment in livestock is generally not affordable, although it is often proposed for viral veterinary diseases as a strategy to fill the gap between the time of the infection and the effective development of the host immune response (3, 4). In contrast, in the case of humans infected or at risk of infection with RVFV, antiviral treatment is fully warranted. Antiviral base and nucleoside analogues effective against RVFV are available (5–7). Among the nucleoside analogs exerting anti-RVFV activity, ribavirin (1-β-d-ribofuranosyl-1-H-1,2,4-triazole-3-carboxamide), favipiravir (6-fluoro-3-hydroxy-2-pyrazinecarboxamide), and more recently BCX4430 [(2S,3S,4R,5R)-2-(4-amino-5H-pyrrolo[3,2-d]pyrimidin-7-yl)-5-(hydroxymethyl)pyrrolidine-3,4-diol] (galidesivir) have been described (6–8). Particularly, favipiravir displays potent antiviral activity against different RNA viruses (9–16). Upon cell intake, favipiravir is converted by cellular enzymes into its active form (favipiravir-4-ribofuranosyl-5-triphosphate), which functions as a purine nucleotide analogue. Favipiravir can act as an RNA chain terminator (17, 18) and as a lethal mutagen for several RNA viruses, including hepatitis C virus (19), foot-and-mouth disease virus (20), West Nile fever virus (21), norovirus (22), and influenza virus (23) among others. Here, we show that the mechanism of action of favipiravir against RVFV in cell culture is due, at least in part, to the accumulation of mutations in the viral genome that leads to a progressive decrease in viable viral progeny. This conclusion is based on a significant increase of mutation frequency measured in a 1,060-bp fragment from the Gc (glycoprotein C)-coding region and a concomitant decrease of specific infectivity associated with virus extinction, supporting the use of favipiravir for the control of emerging RVFV infections in humans.

## RESULTS

### Effect of favipiravir (T-705) on RVFV 56/74 progeny virus yield.

**FIG 1:**
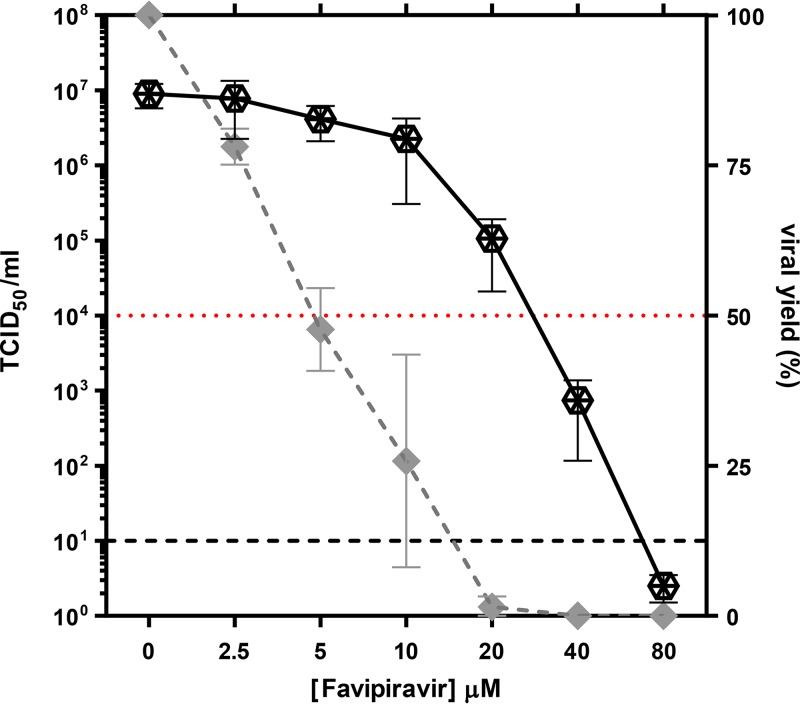
Effect of favipiravir treatment on viral yields in cell culture. Vero cells were pretreated overnight with the indicated concentrations of drug and then infected with RVFV 56/74 at a MOI of 0.1 PFU/cell. Infection proceeded for 72 hpi in the presence of the same concentration of favipiravir. Results are shown either as 50% tissue culture infective dose (TCID_50_) per milliliter (continuous line; left *y* axis) or as percentages of RVFV titer with respect to the one obtained in untreated cells (dashed line; right *y* axis). Values under the limit of sensitivity of the assay (estimated to be 10, indicated with a grid line) were arbitrarily represented as 2. Red line indicates 50%. Error bars denote standard deviation (SD).

The aim of this work was to determine whether favipiravir induces lethal mutagenesis upon RVFV infection in cell culture. We first determined the effect of different concentrations of favipiravir on the yield of RVFV isolate 56/74 in Vero cells. The 50% inhibitory concentration (IC_50_) (concentration required for a 50% decrease in the production of infectious progeny) was calculated to be 6.22 ± 1.5 μM (average of 3 determinations) (Fig. 1). Since the 50% cytotoxic concentration (CC_50_) of T-705 for Vero cells is >10,000 μM (21), the therapeutic index (TI) for RVFV (TI = CC_50_/IC_50_) is >1,600.

**FIG 2:**
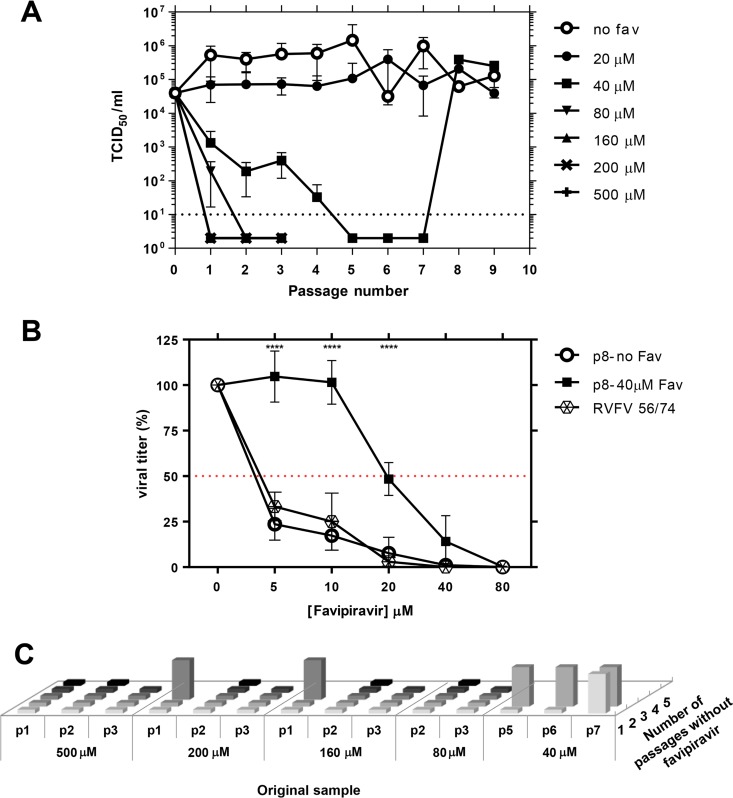
Serial passages of RVFV in Vero cells in the absence or presence of favipiravir. (A) Viral titers of RVFV after each passage in the absence or presence of different concentrations of favipiravir (T-705) as indicated. For concentrations higher than 80 μM, only 3 passages were performed. Viral titers in the supernatant of infected cells were determined at 72 hpi. Error bars denote SD. (B) Comparison of the viral titers obtained from virus recovered after passage 8 (previously propagated or not with 40 μM favipiravir) and those of parental RVFV 56/74 in the presence of a 5 to 80 μM range of favipiravir concentrations. Titers are represented as percentages with respect to those obtained without drug. Red line indicates 50%. Error bars denote SD. Asterisks denote a *P* value ≤ 0.0001 (p8-no Fav or RVFV 56/74 versus p8-40μM Fav; Student's *t* test). (C) Lack of detectable infectivity after blind passages as a criterion for RVFV extinction. Cell culture supernatants from passages carried out in the presence of favipiravir where no cytopathic effect (CPE) was observed (Fig. 2A; samples whose titers were below the limit of sensitivity of the assay) were subjected to additional passages (up to five) in the absence of drug. Original samples are named according to their passage number in the presence of the indicated favipiravir concentration (in μM). Flattened columns indicate no CPE detection; lifted columns indicate evident CPE.

The RVFV 56/74 isolate was subjected to serial passages in Vero cells in the absence or presence of different concentrations of favipiravir (Fig. 2A). The titration of supernatants indicated loss of infectivity in the passages carried out in the presence of 80 μM favipiravir as well as for higher doses, such as 160, 200, and 500 μM, but not at the lowest concentrations tested. A concentration of 20 μM produced a slight reduction in viral yield during the consecutive passages, while at 40 μM, the progeny production declined progressively and was not detected after the fifth, sixth, and seventh passage. Interestingly, virus yields were recovered at passages 8 and 9. These data suggested the selection of a resistant mutant under certain suboptimal inhibitory concentrations of the drug. To test this hypothesis, viral yields in the presence of different concentrations of favipiravir were analyzed for this virus. As expected from the previous results (Fig. 1), favipiravir concentrations over 5 μM significantly reduced the yield of either the parental or the serially passaged RVFV 56/74 not treated with the drug, while for the virus selected after 8 passages in the presence of 40 μM favipiravir, a 50% reduction in viral yield was achieved at a higher concentration, rendering an IC_50_ of 19.2 μM (Fig. 2B). These results clearly indicated a 3-fold change in susceptibility to the drug when compared to that of the parental strain, although it remained susceptible to higher concentrations of the drug. The characterization of this resistant variant is under investigation.

To confirm virus extinction, cell culture supernatants from passages carried out in the presence of favipiravir where no cytopathic effect (CPE) had been observed were subjected to up to five additional passages in the absence of the drug (Fig. 2C). No infectivity was rescued from samples that were collected after at least two serial passages in the presence of 80 μM favipiravir or higher. Viral infectivity could be recovered from supernatants of cells previously treated with 40 μM favipiravir. These data confirmed the previous observation, indicating that the deleterious effect on virus propagation at this concentration was not complete.

### Analysis of RVFV passaged in absence or presence of favipiravir.

**FIG 3:**
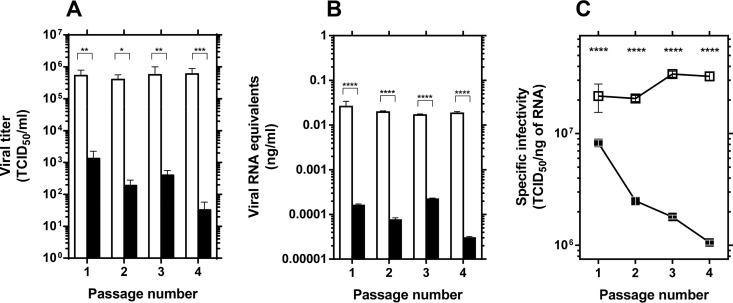
Effect of favipiravir in the specific activity of RVFV. (A) Viral titers of RVFV after each passage (*n* = 4) in the absence (white columns) or presence (black columns) of 40 μM favipiravir (redrawn from data shown in Fig. 2A). (B) Quantification by qRT-PCR (in triplicate) of RNA extracted from the supernatants of untreated (white columns) or 40 μM favipiravir-treated (black columns) Vero cells after each passage (*n* = 4). (C) Specific infectivity upon passage of RVFV in the absence (open symbols) or presence (closed symbols) of 40 μM favipiravir. Values correspond to the ratio between infectivity (A) and the amount of viral RNA (B). Error bars denote SD. Statistically significant differences are indicated by asterisks (*, *P* ≤ 0.05; **, *P* ≤ 0.01; ***, *P* ≤ 0.005; ****, *P* ≤ 0.0001; Student's *t* test).

Decrease of specific infectivity, broadening of the mutant spectrum, and invariance of the consensus sequence of the viral population are diagnostic of lethal mutagenesis (19–21, 23–28). To explore these parameters, RVFV populations at passages 1 to 4 in the absence or presence of 40 μM favipiravir were examined. Extracellular viral titers and viral RNA decreased significantly in the presence of favipiravir (Fig. 3). The changes in infectivity and viral RNA production resulted in a gradual decrease of specific infectivity in the populations passaged in the presence of favipiravir (up to 8-fold in four passages) but not in its absence (*P* < 0.0001 for passages 1 to 4; *t* test). Thus, the first diagnosis criterion of lethal mutagenesis is fulfilled for favipiravir acting on RVFV.

**FIG 4:**
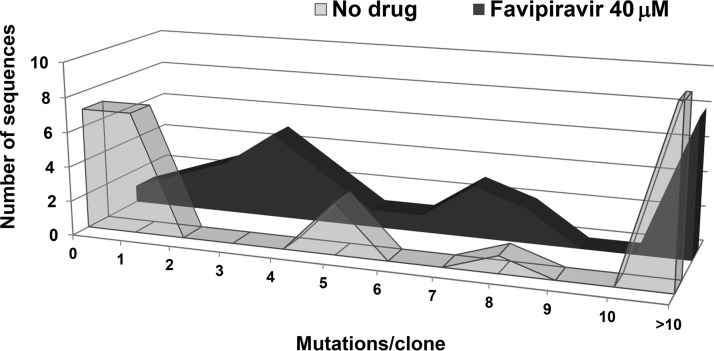
Distribution of mutations among individual clones. Plot of the distribution of the number of mutations per molecular clone in the RVFV populations passaged in the absence or presence of 40 μM favipiravir.

To examine whether the decrease in progeny production was associated with increased mutagenesis of the viral RNA, total RNA from the cell culture supernatant of passage 4 in the absence or presence of 40 μM favipiravir was extracted, and a 1.1-kb DNA fragment encoding a glycoprotein Gc region comprising nucleotides 2091 to 3223 (numbering according to GenBank sequence of RVFV strain SA-75; GenBank accession number DQ380189) was amplified by reverse transcriptase PCR (RT-PCR) and subjected to molecular cloning and nucleotide sequencing. For each condition (with/without drug), a total of 29 clones were analyzed, corresponding to 31,610 nucleotides (nt) sequenced. The results (Table 1) indicated a significant 2.5-fold increase of minimum mutation frequency associated with replication in the presence of favipiravir (*P* < 0.0001; χ^2^ test). Of the total 142 different mutations identified in the populations analyzed, 89 were detected only in the population passaged in the presence of favipiravir, 4 were detected only in the population passaged in the absence of favipiravir, and 49 were common in the two populations (indicated in Table S1 in the supplemental material). Once the common mutations are excluded, the minimum mutation frequencies are 1.3 × 10^−4^ and 2.8 × 10^−3^ in the absence and presence of favipiravir, respectively, and the difference is statistically significant (*P* < 0.0001; χ^2^ test). The distribution of the number of Gc sequences was analyzed as a function of the number of mutations per clone (Fig. 4). While the viral population that was passaged in the absence of drug yielded 7 clones without mutations and 7 clones with only one mutation (24.1% in both cases), only 1 clone without mutations and 2 with one mutation were retrieved from the population that was passaged in the presence of favipiravir (1/29, 3.4%; 2/29, 6.9%, respectively). Finally, the consensus genomic nucleotide sequence (analyzed at passage 4) did not change as a result of replication in the presence of favipiravir, an observation already found during lethal mutagenesis of other RNA viruses (19, 27–30).

**TABLE 1:** Mutant spectrum analysis by molecular cloning and Sanger sequencing of the glycoprotein Gc-coding region of RVFV passaged 4 times in Vero cells

RVFV population^*a*^	No. of nucleotides analyzed (clones/haplotypes)^*b*^	No. different (total) mutations^*c*^	Minimum mutation frequency^*d*^	Maximum mutation frequency^*e*^	No. different synonymous (nonsynonymous) mutations	Total no. synonymous (nonsynonymous) mutations
56/74 p4, no drug	31,610 (29/21)	53 (375)	1.7 × 10^−3^	1.2 × 10^−2^	48 (5)	354 (21)
56/74 p4 + favipiravir	31,610 (29/29)	138 (348)	4.4 × 10^−3^	1.1 × 10^−2^	85 (53)	284 (64)

a The origin and passage history of the viral populations analyzed is described in Fig. 2 and in Materials and Methods.

b The genomic region analyzed spans residues 2110 to 3199 of the glycoprotein Gc-coding region; the residue numbering is that of the SA-75 genome. The values in parenthesis indicate the number of clones analyzed followed by the number of haplotypes (number of different RNA sequences).

c The number of different and total mutations were counted relative to the consensus sequence of the corresponding population. Different and total mutations were used to calculate the minimum and maximum mutation frequency, respectively.

d Data represent the average number of different mutations per nucleotide in the mutant spectrum relative to the consensus sequence of the corresponding population. The results indicate a significant 2.5-fold increase of minimum mutation frequency associated with replication in the presence of favipiravir (*P* < 0.0001; χ^2^ test).

e Data represent the average number of total mutations per nucleotide in the mutant spectrum relative to the consensus sequence of the corresponding population.

### Analysis of mutation types.

**FIG 5:**
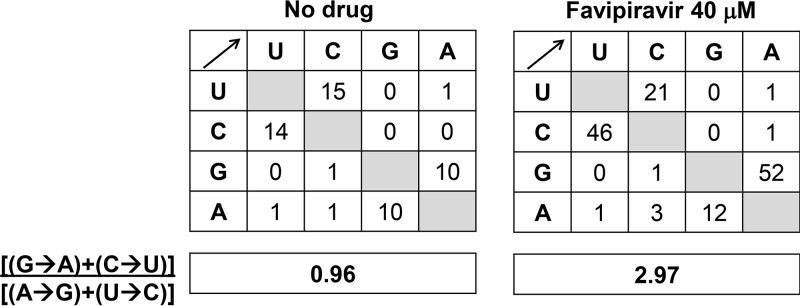
Mutational spectrum induced by favipiravir on RVFV. Matrices of mutation types found in the glycoprotein Gc-coding region of RVFV passaged 4 times in Vero cells in the absence or presence of 40 μM favipiravir. The boxes below each matrix quantify the mutational bias according to the ratio shown on the left.

In order to analyze whether passage of RVFV in the presence of favipiravir resulted in any deviation of mutation types, we compared the mutation matrices in the mutant spectrum of the populations passaged in the absence or presence of the drug. The results indicate a predominance of G to A and C to U transitions associated with replication in the presence of favipiravir as follows: without drug, the number of changes G→A+C→U was 24 (10 + 14), while with favipiravir, it was 98 (52 + 46), thus resulting in a 3-fold increase of the ratio (G→A) + (C→U)/(A→G) + (U→C) (Fig. 5). The difference between the type of mutations was statistically significant (*P* < 0.0001; χ^2^ test) and reveals that favipiravir produces a bias in favor of GA and CU transitions; the transition to transversion ratio was comparable, 12.2 and 18.7 upon passage in the absence and presence of favipiravir, respectively.

These results indicate that favipiravir is a mutagenic agent for RVFV and suggest that its anti-RVFV activity may be exerted at least in part through lethal mutagenesis.

## DISCUSSION

The broad-spectrum antiviral activity of favipiravir has been established with representatives of several families of RNA viruses, so it stands as a promising drug to be used against viral pathogens. In the case of Rift Valley fever, for which no therapeutic or prophylactic treatments are available for humans, favipiravir could be a powerful therapeutic tool to provide immediate treatment to farmers, veterinary personnel, and research personnel at risk of infection after being in contact with the virus or virus-infected animals. Favipiravir was initially considered an inhibitor of viral RNA synthesis, exerted mainly through RNA chain termination (17, 18). However, model studies with several viruses in cell culture and *in vivo* suggest that the antiviral activity of favipiravir may include lethal mutagenesis.

Previous studies have shown that favipiravir treatment of laboratory animals exposed to RVFV increased their survival and counteracted acute or neurological disease syndromes (6, 7). Furthermore, treatment efficacy was enhanced when favipiravir was combined with ribavirin treatment (7). Knowledge of the mechanism of action of this promising drug is important to improve antiviral designs. Our data clearly show at least 2.5-fold increased RVFV mutation frequencies when the virus was propagated in the presence of 40 μM favipiravir, a dose which is 10- to 25-fold lower than the dose proven effective for other RNA viruses tested in a similar way (19–21). The limitation of nonsynonymous mutations introduced during the four passages in the absence of favipiravir (9.4% and 5.6% of the total 53 and 375 for different and total mutations, respectively) is diminished in the passages in the presence of favipiravir (38.4% and 18.4%, respectively), consistent with random introduction of mutations that decrease viral fitness.

One of the most advantageous features of favipiravir is that it shows a high barrier for the generation of resistance mutants (9). Since its activity against human influenza virus was first described (11), only one resistant mutant has been reported (31). Attempts to isolate resistant variants of poliovirus and influenza virus seem to have been unsuccessful (32–34). In addition, influenza virus pairs isolated from patients before and after favipiravir administration showed no changes in the susceptibility to the drug (35). Similar results were reported in norovirus-infected mice after favipiravir treatment (22). Moreover, previous studies reporting favipiravir-mediated viral extinction by lethal mutagenesis did not describe selection of specific mutations or resistant variants (19–21, 23). In our system, 40 μM favipiravir seemed not to lead to the extinction of the virus since after a lag phase of three consecutive blind passages, the presence of CPE was detected again in cultures. The comparison of the IC_50_ value of the virus recovered in supernatant after the lag phase with that of the original samples confirmed the selection of variants with increased favipiravir resistance, suggesting the selection of mutant viruses with increased resistance to the drug. Work is under way in order to characterize this novel resistance variant both at the genomic and phenotypic level *in vitro* and *in vivo*.

The appearance of variants showing decreased susceptibility to the drug has been shown earlier (36), and it highlights the importance of deciphering the exact mechanisms exerted by new promising antiviral drugs, such as favipiravir. In this sense, drug concentration could be proposed as a crucial determinant on the antiviral effect. Thus, between high concentrations, leading to a total abolishment of infectivity, and low concentrations, with none or low effects on viral replication, there might be a critical intermediate concentration that, even though forcing the virus into lethal mutagenesis, promotes the selection for drug-resistant variants before the virus is extinguished. Nonetheless, it is worth noting that other studies found that drug concentrations close to the IC_50_ led to viral extinction of influenza virus without the emergence of variants (23), perhaps indicating that other unknown factors play a role the inhibitory mechanism of T-705. Still, the antiviral activity mechanism of favipiravir is not fully understood. Most nucleoside analogues used as antiviral agents display several mechanisms of activity (37–39). This is also the case for favipiravir, with dual chain terminating and mutagenic activity by ambiguous base pairing as well as a possible role in modulation of innate immunity (10).

In summary, what our results suggest is that favipiravir can act as a lethal mutagen for RVFV, but they do not exclude that other mechanisms, alone or together with lethal mutagenesis, are required for virus extinction.

## MATERIALS AND METHODS

### Cells, viruses, and infections.

Vero cells (ATCC CCL-81) were grown in Dulbecco’s modiﬁed Eagle’s medium (DMEM) supplemented with 5% to 10% fetal calf serum (FCS) and l-glutamine (2 mM), penicillin (100 U/ml), and streptomycin (100 μg/ml) in a humid atmosphere of 5% CO_2_ at 37°C.

The virus used in this study originates from a sheep experimentally infected in our laboratory (40) with RVFV isolate 56/74 (41, 42). The virus was reisolated from infected sheep plasma by culturing in a C6/36 mosquito cell line (ATCC CRL-1660). The infection of cells in the absence of drugs and the assays for titration of infectivity in liquid medium to determine tissue culture infective doses (TCID) were performed as previously described (43). Titers were determined according to reference 44. Assays to quantify PFU were done in semisolid medium including 1% carboxymethylcellulose (CMC; Sigma). Monolayers were fixed and stained 3 to 5 days postinfection.

### Infection in the presence of favipiravir.

Favipiravir T-705 (Atomax Chemicals Co. Ltd., China) was dissolved in dimethyl sulfoxide (DMSO) at a concentration of 100 mM, aliquoted, and stored at −70°C. For use in the experiments, the solution was thawed and diluted in DMEM to reach the desired concentration indicated in each experiment. Cells were pretreated with favipiravir 14 h prior to infection, the virus was adsorbed for 1 h to cells in the absence of drug, and then infection continued in the presence of the same drug concentration.

Serial passages in the presence of favipiravir were performed by infecting Vero cell monolayers seeded on M6 plates in 2 ml of cell culture medium; 0.5 ml of the supernatant was collected at 72 h postinfection (hpi) and used for the next infection. The first passage was carried out at a multiplicity of infection (MOI) of 0.1 PFU/cell (4 × 10^5^ cells infected with 4 × 10^4^ PFU of virus). Mock-infected cells were maintained in parallel to control for the absence of contaminating virus. When no cytopathic effect (CPE) was observed after several passages in the presence of favipiravir, the cell culture supernatant was used to perform up to 5 blind passages in the absence of drug to ascertain that no infectious virus could be recovered. Lack of detectable CPE before and after blind passages was considered as criterion of RVFV extinction. The IC_50_ of T-705 was calculated as described previously (19).

### RNA extraction, RT-PCR, and nucleotide sequencing.

RNA was extracted from the supernatants of infected cells using the Speedtools RNA virus extraction kit (Biotools B&M Labs, S.A., Madrid, Spain) according to the manufacturer’s instructions. RT-PCR was performed using SuperScript IV reverse transcriptase and Phusion high-fidelity DNA polymerase (Thermo Scientific). cDNA was obtained by retrotranscription from RVFV M-segment RNA using primer MRV1ag (5′-CAAATGACTACCAGTCAGC-3′; positions 772 to 790; antigenomic orientation) as described previously (45). The cDNA was then amplified by PCR using oligonucleotides startGc forward (5′-ACTCGCATTGTCGACGTTTT) (positions 2091 to 2109; genomic orientation) and sm3 reverse (5′-GGATTAAGGAAGCGGGAAAAGCCC) (positions 3200 to 3223; antigenomic orientation). The amplified cDNA is a 1,100-nt fragment that corresponds to the amino-terminal half of glycoprotein Gc, and it was chosen because it is highly conserved in RVFV (46). Amplifications in the absence of RNA were carried out in parallel to ascertain the absence of contaminating templates. For the analysis of mutant spectra, amplifications were carried out with template preparations diluted 1:10, 1:100, and 1:1,000. When amplification of the 1:100 diluted template yielded a detectable DNA fragment, cloning was performed with the amplified fragment obtained with the undiluted template, as described previously (47). This procedure ensures an excess of template viral RNA molecules, thus avoiding redundant amplifications of the same initial RNA molecules. Amplified DNA fragments were then ligated to a pJET2.1 plasmid (Thermo Scientific) and the products transformed into Escherichia coli DH5α. The pJET2.1 vector contains the lethal gene *eco47IR*, enabling positive selection of recombinant plasmids. Plasmid DNA from individual bacterial colonies was purified and subjected to nucleotide sequencing, as previously described (48).

### Quantification of RVFV RNA.

For the calculation of specific infectivity (the ratio of infectivity to viral RNA in a virus sample), a real-time quantitative one-step PCR (RT-qPCR) of a fragment within L-segment RNA was carried out using RNA HighScript master mix (Biotools B&M), according to the manufacturer’s instructions. Primers used were L-forward (5′-TTCTTTGCTTCTGATACCCTCTG-3′) (5′ position 2872; sense orientation) and L-reverse (5′-GTTCCACTTCCTTGCATCATCTG) (5′ position 3006; antisense orientation) that yield a 135-bp fragment within the RdRp-coding region (42). Detection was achieved by an L-segment-specific hydrolysis probe (5′-^[6-FAM]^TTGCACAAGTCCACACAGGCCCCT^[BHQ1]^-3′). Quantification of RNA was relative to a standard curve obtained with known amounts of RVFV RNA, synthesized by *in vitro* transcription of an NdeI-linearized pGEM-T plasmid containing the 135-bp cDNA target fragment using the T7 RiboMax *in vitro* transcription kit (Promega). Negative controls (without template RNA and RNA from mock-infected cells) were run in parallel with each amplification reaction to ascertain absence of contamination with undesired templates.

### Statistical analysis.

Data analysis was performed using GraphPad Prism software version 6.** **

## Supplementary Material

Supplemental file 1

## References

[1] Chevalier V 2013 Relevance of Rift Valley fever to public health in the European Union. Clin Microbiol Infect 19:705–708. doi:10.1111/1469-0691.12163. 23517372

[2] Rolin AI, Berrang-Ford L, Kulkarni MA 2013 The risk of Rift Valley fever virus introduction and establishment in the United States and European Union. Emerg Microbes Infect 2:e81. doi:10.1038/emi.2013.81. 26038446 PMC3880870

[3] Dal Pozzo F, Thiry E 2014 Antiviral chemotherapy in veterinary medicine: current applications and perspectives. Rev Sci Tech 33:791–801. doi:10.20506/rst.33.3.2318. 25812204

[4] De Vleeschauwer AR, Lefrebvre DJ, De Clercq K 2017 Antiviral therapies for foot-and-mouth disease, p 357–384. *In* Sobrino F, Domingo E (ed), Foot-and-mouth disease virus current research and emerging trends. Caister Academic Press, Norfolk, UK.

[5] Gowen BB, Wong MH, Jung KH, Sanders AB, Mendenhall M, Bailey KW, Furuta Y, Sidwell RW 2007 In vitro and in vivo activities of T-705 against arenavirus and bunyavirus infections. Antimicrob Agents Chemother 51:3168–3176. doi:10.1128/AAC.00356-07. 17606691 PMC2043187

[6] Caroline AL, Powell DS, Bethel LM, Oury TD, Reed DS, Hartman AL 2014 Broad spectrum antiviral activity of favipiravir (T-705): protection from highly lethal inhalational Rift Valley fever. PLoS Negl Trop Dis 8:e2790. doi:10.1371/journal.pntd.0002790. 24722586 PMC3983105

[7] Scharton D, Bailey KW, Vest Z, Westover JB, Kumaki Y, Van Wettere A, Furuta Y, Gowen BB 2014 Favipiravir (T-705) protects against peracute Rift Valley fever virus infection and reduces delayed-onset neurologic disease observed with ribavirin treatment. Antiviral Res 104:84–92. doi:10.1016/j.antiviral.2014.01.016. 24486952 PMC3975078

[8] Westover JB, Mathis A, Taylor R, Wandersee L, Bailey KW, Sefing EJ, Hickerson BT, Jung KH, Sheridan WP, Gowen BB 2018 Galidesivir limits Rift Valley fever virus infection and disease in Syrian golden hamsters. Antiviral Res 156:38–45. doi:10.1016/j.antiviral.2018.05.013. 29864447 PMC6035881

[9] Delang L, Abdelnabi R, Neyts J 2018 Favipiravir as a potential countermeasure against neglected and emerging RNA viruses. Antiviral Res 153:85–94. doi:10.1016/j.antiviral.2018.03.003. 29524445

[10] de la Torre JC 2018 Extending the antiviral value of favipiravir. J Infect Dis 218:509–511. doi:10.1093/infdis/jiy153. 29762707

[11] Furuta Y, Takahashi K, Fukuda Y, Kuno M, Kamiyama T, Kozaki K, Nomura N, Egawa H, Minami S, Watanabe Y, Narita H, Shiraki K 2002 In vitro and in vivo activities of anti-influenza virus compound T-705. Antimicrob Agents Chemother 46:977–981. doi:10.1128/aac.46.4.977-981.2002. 11897578 PMC127093

[12] Furuta Y, Komeno T, Nakamura T 2017 Favipiravir (T-705), a broad spectrum inhibitor of viral RNA polymerase. Proc Jpn Acad Ser B Phys Biol Sci 93:449–463. doi:10.2183/pjab.93.027. PMC5713175 28769016

[13] Qiu L, Patterson SE, Bonnac LF, Geraghty RJ 2018 Nucleobases and corresponding nucleosides display potent antiviral activities against dengue virus possibly through viral lethal mutagenesis. PLoS Negl Trop Dis 12:e0006421. doi:10.1371/journal.pntd.0006421. 29672522 PMC5929572

[14] Guedj J, Piorkowski G, Jacquot F, Madelain V, Nguyen THT, Rodallec A, Gunther S, Carbonnelle C, Mentre F, Raoul H, de Lamballerie X 2018 Antiviral efficacy of favipiravir against Ebola virus: a translational study in cynomolgus macaques. PLoS Med 15:e1002535. doi:10.1371/journal.pmed.1002535. 29584730 PMC5870946

[15] Bassi MR, Sempere RN, Meyn P, Polacek C, Arias A 2018 Extinction of Zika virus and Usutu virus by lethal mutagenesis reveals different patterns of sensitivity to three mutagenic drugs. Antimicrob Agents Chemother 62:e00380-18. doi:10.1128/AAC.00380-18. 29914957 PMC6125542

[16] Goldhill DH, Langat P, Xie H, Galiano M, Miah S, Kellam P, Zambon M, Lackenby A, Barclay WS 2019 Determining the mutation bias of favipiravir in influenza virus using next-generation sequencing. J Virol 93:e01217-18. doi:10.1128/JVI.01217-18. 30381482 PMC6321902

[17] Jin Z, Smith LK, Rajwanshi VK, Kim B, Deval J 2013 The ambiguous base-pairing and high substrate efficiency of T-705 (favipiravir) ribofuranosyl 5'-triphosphate towards influenza A virus polymerase. PLoS One 8:e68347. doi:10.1371/journal.pone.0068347. 23874596 PMC3707847

[18] Sangawa H, Komeno T, Nishikawa H, Yoshida A, Takahashi K, Nomura N, Furuta Y 2013 Mechanism of action of T-705 ribosyl triphosphate against influenza virus RNA polymerase. Antimicrob Agents Chemother 57:5202–5208. doi:10.1128/AAC.00649-13. 23917318 PMC3811313

[19] de Avila AI, Gallego I, Soria ME, Gregori J, Quer J, Esteban JI, Rice CM, Domingo E, Perales C 2016 Lethal mutagenesis of hepatitis C virus induced by favipiravir. PLoS One 11:e0164691. doi:10.1371/journal.pone.0164691. 27755573 PMC5068784

[20] de Avila AI, Moreno E, Perales C, Domingo E 2017 Favipiravir can evoke lethal mutagenesis and extinction of foot-and-mouth disease virus. Virus Res 233:105–112. doi:10.1016/j.virusres.2017.03.014. 28322918

[21] Escribano-Romero E, Jimenez de Oya N, Domingo E, Saiz JC 2017 Extinction of West Nile virus by favipiravir through lethal mutagenesis. Antimicrob Agents Chemother 61:e01400-17. doi:10.1128/AAC.01400-17. PMC5655064 28848019

[22] Arias A, Thorne L, Goodfellow I 2014 Favipiravir elicits antiviral mutagenesis during virus replication in vivo. Elife 3:e03679. doi:10.7554/eLife.03679. 25333492 PMC4204012

[23] Baranovich T, Wong SS, Armstrong J, Marjuki H, Webby RJ, Webster RG, Govorkova EA 2013 T-705 (favipiravir) induces lethal mutagenesis in influenza A H1N1 viruses in vitro. J Virol 87:3741–3751. doi:10.1128/JVI.02346-12. 23325689 PMC3624194

[24] Loeb LA, Essigmann JM, Kazazi F, Zhang J, Rose KD, Mullins JI 1999 Lethal mutagenesis of HIV with mutagenic nucleoside analogs. Proc Natl Acad Sci U S A 96:1492–1497. doi:10.1073/pnas.96.4.1492. 9990051 PMC15492

[25] Sierra S, Davila M, Lowenstein PR, Domingo E 2000 Response of foot-and-mouth disease virus to increased mutagenesis: influence of viral load and fitness in loss of infectivity. J Virol 74:8316–8323. doi:10.1128/jvi.74.18.8316-8323.2000. 10954530 PMC116341

[26] Ortega-Prieto AM, Sheldon J, Grande-Perez A, Tejero H, Gregori J, Quer J, Esteban JI, Domingo E, Perales C 2013 Extinction of hepatitis C virus by ribavirin in hepatoma cells involves lethal mutagenesis. PLoS One 8:e71039. doi:10.1371/journal.pone.0071039. 23976977 PMC3745404

[27] Sánchez-Campos S, Domínguez-Huerta G, Díaz-Martínez L, Tomás DM, Navas-Castillo J, Moriones E, Grande-Pérez A 2018 Differential shape of geminivirus mutant spectra across cultivated and wild hosts with invariant viral consensus sequences. Front Plant Sci 9:932. doi:10.3389/fpls.2018.00932. 30013589 PMC6036239

[28] Grande-Pérez A, Gómez-Mariano G, Lowenstein PR, Domingo E 2005 Mutagenesis-induced, large fitness variations with an invariant arenavirus consensus genomic nucleotide sequence. J Virol 79:10451–10459. doi:10.1128/JVI.79.16.10451-10459.2005. 16051837 PMC1182645

[29] Domingo E 2016 Virus as populations. Elsevier Academic Press, Amsterdam, Netherlands.

[30] González-López C, Gómez-Mariano G, Escarmís C, Domingo E 2005 Invariant aphthovirus consensus nucleotide sequence in the transition to error catastrophe. Infect Genet Evol 5:366–374. doi:10.1016/j.meegid.2005.05.001. 16002345

[31] Goldhill DH, Te Velthuis AJW, Fletcher RA, Langat P, Zambon M, Lackenby A, Barclay WS 2018 The mechanism of resistance to favipiravir in influenza. Proc Natl Acad Sci U S A 115:11613–11618. doi:10.1073/pnas.1811345115. 30352857 PMC6233120

[32] Daikoku T, Yoshida Y, Okuda T, Shiraki K 2014 Characterization of susceptibility variants of influenza virus grown in the presence of T-705. J Pharmacol Sci 126:281–284. doi:10.1254/jphs.14156SC. 25296868

[33] Daikoku T, Mizuguchi M, Obita T, Yokoyama T, Yoshida Y, Takemoto M, Shiraki K 2018 Characterization of susceptibility variants of poliovirus grown in the presence of favipiravir. J Microbiol Immunol Infect 51:581–586. doi:10.1016/j.jmii.2017.03.004. 28709841

[34] Vanderlinden E, Vrancken B, Van Houdt J, Rajwanshi VK, Gillemot S, Andrei G, Lemey P, Naesens L 2016 Distinct effects of T-705 (favipiravir) and ribavirin on influenza virus replication and viral RNA synthesis. Antimicrob Agents Chemother 60:6679–6691. doi:10.1128/AAC.01156-16. 27572398 PMC5075073

[35] Takashita E, Fujisaki S, Shirakura M, Nakamura K, Kishida N, Kuwahara T, Shimazu Y, Shimomura T, Watanabe S, Odagiri T 2016 Influenza A(H1N1)pdm09 virus exhibiting enhanced cross-resistance to oseltamivir and peramivir due to a dual H275Y/G147R substitution, Japan, March 2016. Euro Surveill 21:pii=30258. 10.2807/1560-7917.ES.2016.21.24.30258 27336226

[36] Delang L, Segura Guerrero N, Tas A, Querat G, Pastorino B, Froeyen M, Dallmeier K, Jochmans D, Herdewijn P, Bello F, Snijder EJ, de Lamballerie X, Martina B, Neyts J, van Hemert MJ, Leyssen P 2014 Mutations in the chikungunya virus non-structural proteins cause resistance to favipiravir (T-705), a broad-spectrum antiviral. J Antimicrob Chemother 69:2770–2784. doi:10.1093/jac/dku209. 24951535

[37] Beaucourt S, Vignuzzi M 2014 Ribavirin: a drug active against many viruses with multiple effects on virus replication and propagation. Molecular basis of ribavirin resistance. Curr Opin Virol 8:10–15. doi:10.1016/j.coviro.2014.04.011. 24846716 PMC7102760

[38] Parker WB 2005 Metabolism and antiviral activity of ribavirin. Virus Res 107:165–171. doi:10.1016/j.virusres.2004.11.006. 15649562

[39] Snell NJ 2001 Ribavirin–current status of a broad spectrum antiviral agent. Expert Opin Pharmacother 2:1317–1324. doi:10.1517/14656566.2.8.1317. 11585000

[40] Lorenzo G, Lopez-Gil E, Ortego J, Brun A 2018 Efficacy of different DNA and MVA prime-boost vaccination regimens against a Rift Valley fever virus (RVFV) challenge in sheep 12 weeks following vaccination. Vet Res 49:21. doi:10.1186/s13567-018-0516-z. 29467018 PMC5822472

[41] Barnard BJ, Botha MJ 1977 An inactivated rift valley fever vaccine. J S Afr Vet Assoc 48:45–48. 874947

[42] Busquets N, Xavier F, Martín-Folgar R, Lorenzo G, Galindo-Cardiel I, del Val BP, Rivas R, Iglesias J, Rodríguez F, Solanes D, Domingo M, Brun A 2010 Experimental infection of young adult European breed sheep with Rift Valley fever virus field isolates. Vector Borne Zoonotic Dis 10:689–696. doi:10.1089/vbz.2009.0205. 20854022

[43] Lorenzo G, Martin-Folgar R, Hevia E, Boshra H, Brun A 2010 Protection against lethal Rift Valley fever virus (RVFV) infection in transgenic IFNAR^−/−^ mice induced by different DNA vaccination regimens. Vaccine 28:2937–2944. doi:10.1016/j.vaccine.2010.02.018. 20188678

[44] Lindenbach BD 2009 Measuring HCV infectivity produced in cell culture and in vivo. Methods Mol Biol 510:329–336. doi:10.1007/978-1-59745-394-3_24. 19009272

[45] Takehara K, Min MK, Battles JK, Sugiyama K, Emery VC, Dalrymple JM, Bishop DH 1989 Identification of mutations in the M RNA of a candidate vaccine strain of Rift Valley fever virus. Virology 169:452–457. doi:10.1016/0042-6822(89)90171-2. 2705307

[46] Bird BH, Khristova ML, Rollin PE, Ksiazek TG, Nichol ST 2007 Complete genome analysis of 33 ecologically and biologically diverse Rift Valley fever virus strains reveals widespread virus movement and low genetic diversity due to recent common ancestry. J Virol 81:2805–2816. doi:10.1128/JVI.02095-06. 17192303 PMC1865992

[47] Airaksinen A, Pariente N, Menendez-Arias L, Domingo E 2003 Curing of foot-and-mouth disease virus from persistently infected cells by ribavirin involves enhanced mutagenesis. Virology 311:339–349. doi:10.1016/S0042-6822(03)00144-2. 12842623

[48] Agudo R, Ferrer-Orta C, Arias A, de la Higuera I, Perales C, Pérez-Luque R, Verdaguer N, Domingo E 2010 A multi-step process of viral adaptation to a mutagenic nucleoside analogue by modulation of transition types leads to extinction-escape. PLoS Pathog 6:e1001072. doi:10.1371/journal.ppat.1001072. 20865120 PMC2928812

