# Supplementary material for: Lethal Mutagenesis of Rift Valley Fever Virus Induced by Favipiravir
Source: Antimicrob Agents Chemother. 2019 Jul 25;63(8):e00669-19. doi: 10.1128/AAC.00669-19 (PMC6658772; doi:10.1128/AAC.00669-19)
Supplement: Supplemental file 1 [file AAC.00669-19-s0001.pdf]

Table S1. Mutations, corresponding amino acid and point accepted mutation (PAM) of the glycoprotein Gc coding region in the mutant spectra RVFV subjected to four passages in the absence or presence of 40 µM Favipiravir (T-705)

No drug			40 µM favipiravir		
**Mutation[a]**	**Amino acid substitution**	**PAM250[b]**	**Mutation[a]**	**Amino acid substitution**	**PAM250[b]**
G2073A (8)	-	-	**G2045A**	S698N	1
T2079C (8)	-	-	**C2046T**	-	
A2094G (7)	-	-	**C2055T**	-	
G2112A (7)	-	-	**A2056C**	T702P	0
A2148G (8)	-	-	**C2057T**	T702I	0
G2154A (8)	-	-	**C2058T**	-	
T2172C (8)	-	-	**C2060T**	T703I	0
A2181G (8)	-	-	**G2063A**	C704Y	0
C2184T (8)	-	-	**C2064T**	-	
C2259T (9)	-	-	**C2066T**	S705F	-3
**C2283T**	-	-	G2073A (4)	-	
T2292C (9)	-	-	**G2077A**	V709I	4
A2295G (9)	-	-	T2079C (4)	-	
G2328A (9)	-	-	**C2082T**	-	
T2340C (8)	-	-	**A2083C**	T711P	0
G2349A (8)	-	-	**G2088A**	-	
A2382G (8)	-	-	A2094G (4)	-	
T2388C (8)	-	-	**G2097A**	-	
G2418A (7)	-	-	**C2103T**	-	
C2427T (6)	-	-	**C2105T**	T718I	0
C2466T (6)	-	-	**T2111C**	L720S	-3
T2478C (6)	-	-	G2112A (5)	-	
T2502C (6)	-	-	**G2131A**	G727R	-3
C2514T (6)	-	-	**G2133A**	-	
G2531C (6)	S860T	1	**C2141T**	A730V	0
C2541T (6)	-	-	**T2145C**	-	
A2544T (6)	-	-	A2148G (4)	-	
T2575C (7)	-	-	G2154A (5)	-	
T2640C (8)	-	-	**G2160A**	-	
T2655C (8)	-	-	T2172C (3)	-	
T2664C (7)	-	-	A2181G (3)	-	
T2754C (8)	-	-	C2184T (3)	-	
**C2759T** (3)	S936L	-3	**G2219A**	C756Y	0
G2763A (6)	-	-	**G2223A**	-	
C2766T (6)	-	-	**G2227A**	G759S	1

C2808T (8)	-	-	**C2229T**	-	
C2814T (8)	-	-	C2259T (6)	-	
A2838G (7)	-	-	**C2271T**	-	
T2868C (7)	-	-	T2292C (6)	-	
T2889C (8)	-	-	A2295G (6)	-	
**G2911A**	E987K	0	**G2307A**	-	
A2913C (9)	E987D	3	**T2320C**	S790P	1
**A2933G** (2)	K994R	3	**G2325A**	W791STOP	
A2976G (9)	-	-	G2328A (6)	-	
T2988A (10)	-	-	T2340C (6)	-	
C3030T (7)	-	-	**C2345T**	A798V	0
A3033G (7)	-	-	G2349A (5)	-	
C3057T (7)	-	-	**G2359A**	V803I	4
T3075C (8)	-	-	**G2364A**	-	
G3084A (8)	-	-	**C2376T**	-	
A3087G (8)	-	-	A2382G (4)	-	
G3093A (8)	-	-	**G2385A**	-	
C3096T (6)	-	-	T2388C (3)	-	
			**G2403A**	-	
			**G2405A**	C818Y	0
			**G2416A**	G822R	-3
			G2418A (5)	-	
			**G2424A**	-	
			C2427T (4)	-	
			**C2439T**	-	
			**G2457A**	-	
			C2466T (4)	-	
			T2478C (5)	-	
			**C2489T**	A846V	0
			T2502C (5)	-	
			C2514T (5)	-	
			G2531C (5)	S860T	1
			**C2533T**	-	
			C2541T (6)	-	
			**C2543T**	T864I	0
			A2544T (6)	-	
			**G2554A**	G868S	1
			T2575C (8)	-	
			**G2578A**	G876R	-3
			**C2592T**	-	

			**T2597C**	F882S	-3
			**C2600T**	T883I	0
			**C2604T**	-	
			**G2614A**	V888I	4
			**C2626T** (2)	-	
			**G2628A**	-	
			**C2633T**	A894V	0
			T2640C (8)	-	
			T2655C (8)	-	
			**C2663T**	S904F	-3
			T2664C (8)	-	
			**C2693T**	A914V	0
			**C2708T**	P919L	-3
			**C2714T**	S921L	-3
			**C2723T**	P924L	-3
			**G2727A**	-	
			**A2730G**	-	
			**G2731A**	G927A	1
			**C2736T**	-	
			**G2743A**	E931K	0
			T2754C (6)	-	
			G2763A (6)	-	
			C2766T (6)	-	
			C2808T (6)	-	
			C2814T (6)	-	
			**G2829A**	M959I	2
			A2838G (7)	-	
			**G2844A**	-	
			**C2847T**	-	
			**C2849T**	T966I	0
			T2868C (7)	-	
			**G2872A**	V974I	4
			**T2879C**	F976S	-3
			**G2881A** (2)	E977K	0
			**G2886A**	-	
			**G2888A**	G979D	1
			T2889C (7)	-	
			**G2906A**	R985K	3
			**G2907A**	-	
			**A2908G**	N986D	2

			A2913C (7)	E987D	3
			**C2918A**	T989N	0
			**C2924T**	A991V	0
			**G2935A**	G995R	-3
			**G2966A**	G1005D	1
			A2976G (5)	-	
			**T2988C**	-	
			**C2989T**	-	
			T2988A (5)	-	
			**G2998A**	D1016N	2
			**G3021A**	-	
			C3030T (5)	-	
			A3033G (5)	-	
			**G3038A**	C1029Y	0
			**A3054G**	-	
			C3057T (5)	-	
			T3075C (4)	-	
			G3084A (4)	-	
			A3087G (4)	-	
			G3093A (4)	-	
			C3096T (4)	-	
			**G3098A**	C1049Y	0
			**G3119A**	G1056E	0

[a]Residue numbering is according to RVFV strain SA-75, accession number DQ380189. The number of clones in which the mutation is found is given in parenthesis. Bold-face indicates mutations found only in absence (first column) or presence (fourth column) of favipiravir.

[b]PAM250 is the amino acid substitution frequency score in which -7 represents minimum acceptability and +7 maximum acceptability, relative to random sequences (1)

References

1. Feng DF, Doolittle RF. 1996. Progressive alignment of amino acid sequences and construction of phylogenetic trees from them. Methods in Enzymol 266:368-82.
